# Supplementary material for: Expression of CTLA-4 and CD86 Antigens and Epstein-Barr Virus Reactivation in Chronic Lymphocytic Leukemia—Any Link with Known Prognostic Factors?
Source: Cancers (Basel). 2022 Jan 28;14(3):672. doi: 10.3390/cancers14030672 (PMC8833759; doi:10.3390/cancers14030672)
Supplement: Supplementary file 1 [file cancers-14-00672-s001.zip › cancers-1495756-supplementary.pdf]

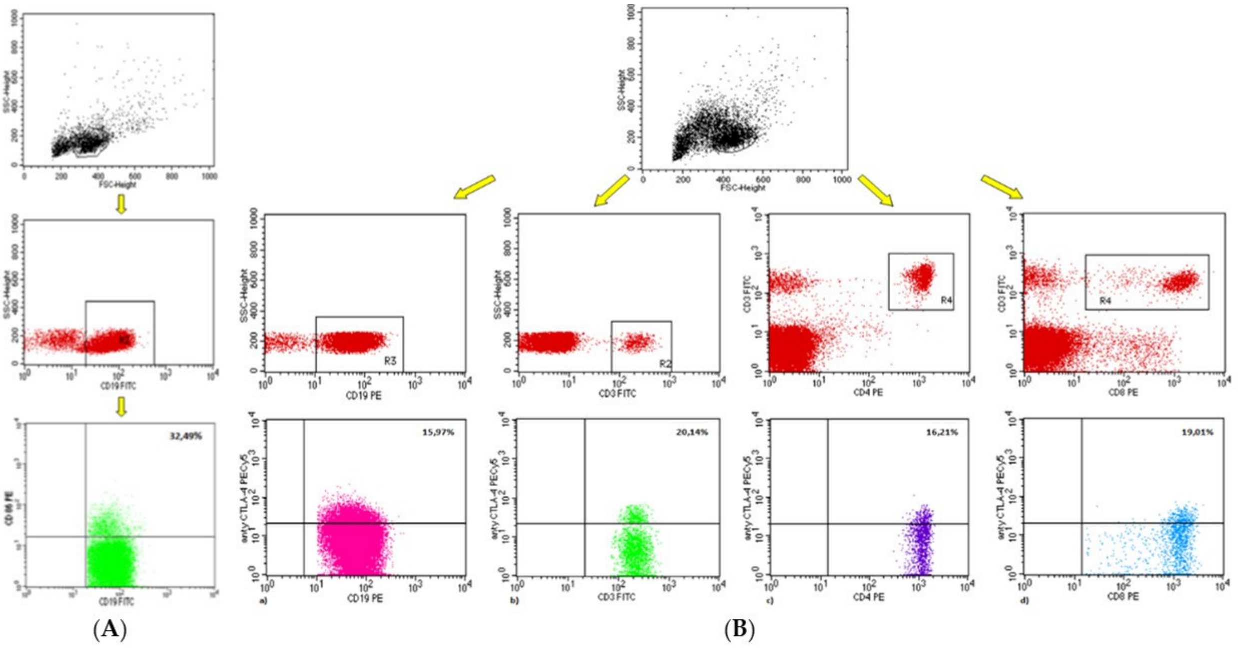

Figure S1. (A): Assessment of the percentage of CD19+ B cells expressing the CD86 antigen in a CLL patient with EBV DNA in PBMC; (**B**): Assessment of the percentage of CD19+ B lymphocytes (**a**), T CD3+ (**b**), T CD4+ (**c**) and T CD8+ (**d**) cells expressing the CTLA-4 antigen in a patient with CLL in which EBV DNA was found.
